# Nurse managers on healthy environments for adolescents living with intellectual disabilities

**DOI:** 10.4102/curationis.v47i1.2592

**Published:** 2024-10-31

**Authors:** Rakgadi G. Malapela

**Affiliations:** 1 Department of Health Studies, School of Social Sciences, University of South Africa, Pretoria, South Africa

**Keywords:** adolescents, healthy environment, intellectual disabilities, nurse managers, perspectives

## Abstract

**Background:**

Providing care for adolescents living with intellectual disabilities (ALWIDs) within healthcare settings is a complex challenge. These adolescents require an environment that is specialised and conducive to their health, alongside tailored care, treatment and rehabilitation services. Nurse managers play a crucial role in supporting these adolescents, particularly given the difficulties parents face in meeting their unique needs.

**Objectives:**

This study aims to explore nurse managers’ perspectives on the creation of a healthy environment for ALWIDs in health facilities.

**Method:**

An explorative qualitative study design was conducted. A purposive sampling method was used to select 13 nurse managers from three institutions relevant to the study’s interests. Nurse managers with over 2 years of direct experience in the care, treatment and rehabilitation of ALWIDs were eligible to participate in the study. Selecting these nurse managers ensured the study captured their firsthand experiences and expertise in managing nursing services. Data were collected through face-to-face and telephonic semi-structured interviews, recorded digitally. Braun and Clarke’s six-step thematic analysis was used for data analysis.

**Results:**

A safe environment; multi-disciplinary team involvement; competent and adequate staff and a conducive physical environment for structural support are beneficial for ALWIDs.

**Conclusion:**

To promote the well-being of ALWIDs, it is essential to establish a spacious and safe environment equipped with sufficient material and non-material resources. Engaging a multi-disciplinary team is vital for addressing their diverse needs.

**Contribution:**

This study provided information on what constitutes a healthy environment for ALWIDs to inform clinical practice, nursing education, policy makers and research.

## Introduction

According to the World Health Organization (WHO 2019), intellectual disability (ID) is characterised by limited cognitive and adaptive functioning. Consequently, adolescents with ID are prone to self-injury or may injure family or peers (Egger 2021). Adolescence, defined as the development stage between childhood and adulthood, spans ages 10–19 years and is a critical period for establishing the foundations of good health (WHO 2024). The United Nations International Children’s Emergency Fund (UNICEF), as cited in Ntshingila et al. (2021), estimates that globally, 1 in 50 parents have a child with ID. In accordance with the *Children’s Act No. 38 of 2005* in South Africa, a child is defined as a person under the age of 18 years. According to Statistics South Africa (SA Stats 2022), the prevalence of disability, inclusive of all disabilities and exclusive of institutionalised population, constitutes a minority at 6%. Adolescents with ID are at higher risk of physical illnesses, and comorbidities, and have higher mortality rates compared to those without ID (Bourke et al. 2017 as cited in Buckley et al. 2020). The Australian Royal has reported significant health disparities, poor access to care and exploitation of people with ID (Disability Royal Commission 2020).

A healthy environment for adolescents living with intellectual disabilities (ALWIDs) contributes positively to their quality of life (Nielsen et al. 2021). This can be achieved by structuring and integrating health promotion services and activities into daily care, which also supports a more individualistic approach. Ntshingila et al. (2021) conclude that most parents face significant challenges when caring for such adolescents, leading to many being admitted to long-term care facilities. These parents experience physical and emotional challenges because of the demands of providing care. Adolescents living with intellectual disabilities require special education, treatment, care, medications, supplementary foods for nutritional problems, transportation, special equipment (such as wheelchair, prosthesis, etc.), physiotherapy and other specialist support, which imposes financial difficulties on families (Balcı et al. 2019; Mert & Köşgeroğlu 2022).

To promote the quality of life for people with ID, the Convention on the Rights of Persons with Disabilities (CRPD) was adopted in 2006 to ensure that all individuals with disabilities have equal access to human rights. Access to a conducive environment is an entitlement that these adolescents should enjoy. This is also supported by the *Constitution of South Africa Act No. 108 of 1996* (Republic of South Africa 1996), the Patients’ Rights Charter (National Department of Health [NDoH] 2002) and the *Mental Health Care Act No. 17 of 2002* (Republic of South Africa 2002) which advocate for a healthy and safe environment. Similarly, according to the United Nations (UN) report (2015), the 2030 Agenda for Sustainable Development Goals in Goal 3, calls for the promotion of the health and well-being of individuals with disabilities.

The constituents of a healthy environment for adolescents with IDs are dependent on the various contexts around the world. Vlot-Van Anrooij et al. (2020a), in a study in the United Kingdom, found four characteristics of a healthy environment for people with ID. These four factors include a standardised physical setting; conditions that allow engagement with society for people with ID; enabling caregivers to implement health promotion activities and enabling caregivers to play a role in society that promotes the well-being of people with ID. In Denmark, Nielsen et al. (2021) posited that one of the critical factors that contributes positively to the well-being of people with ID in long-term care is the competencies, attitudes and values of their nurses and nurse managers. Moreover, Powers et al. (2021) reiterate the issue of the competencies of caregivers and the physical environment of long-term care facilities. They go on to note that the physical environment should have adequate space to promote physical activity. Given the increased risk of physical illness among ALWIDs, nurse managers must facilitate a healthy environment to reduce the occurrence of physical illnesses and injuries.

### Theoretical framework

**FIGURE 1:**
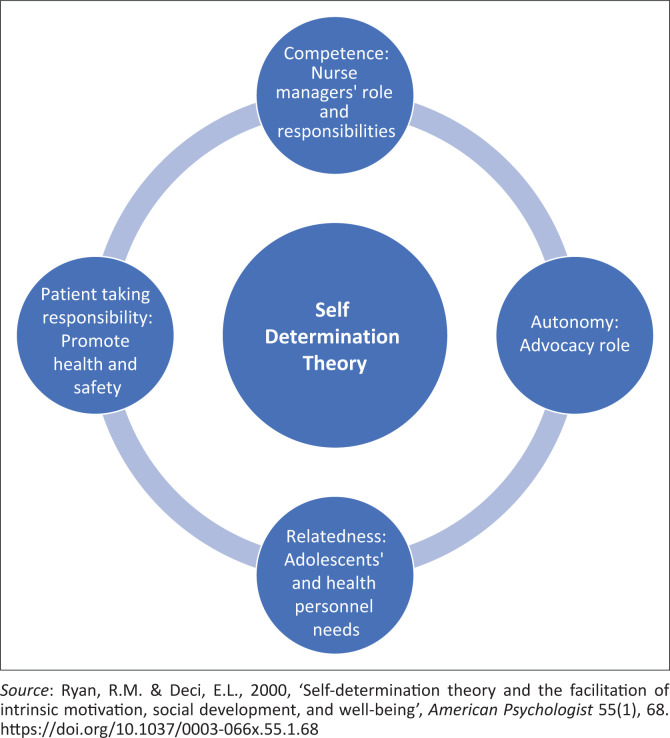
Adapted self-determination theory.

According to Ryan and Deci (2000), Self-Determination Theory (SDT) highlights the fact that individuals can be proactive or passive, depending on the condition under which they are working, and this theory can be applied in different health contexts (Deci & Ryan 2000; Migliorini, Cardinali & Rania 2019). In this study, SDT was used to explore and describe nurse managers’ perspectives on creating a healthy environment for ALWIDs. Furthermore, Ryan and Deci (2017) noted that nurse managers need to take autonomous decisions about more activities that promote healthy outcomes. The SDT will be useful to determine what should be considered to achieve autonomy, competence, relatedness and patient-taking responsibility (Refer to Figure 1 for the adapted SDT).

Universal needs play a major role in providing optimal patient care and meeting the psychological needs of adolescents with IDs according to SDT (Deci & Ryan 2008; Ohajunwa & Mji 2018; Ryan & Deci 2000). This includes the following:

- *Competence:* This refers to the role and responsibilities that a nurse manager needs to undertake to reach the desired goal and promote quality patient care among ALWIDs. This requires nurse managers to take the advocacy role to promote a conducive environment that is favourable for these adolescents, their families and healthcare providers.
- *Autonomy:* This pertains to nurse managers’ capacity to express their viewpoints and make decisions regarding the conditions that contribute to a healthy environment for ALWIDs.
- *Relatedness:* This means considering what is needed to maintain a good working relationship with health personnel. The nurse managers also need to consider the needs of the health personnel to promote a conducive environment for ALWIDs and health personnel.
- *Patient-taking responsibility*. When nurse managers take responsibility for making decisions that promote the well-being of ALWIDs, it empowers the patients to assume responsibility for their own health and safety.

According to the literature reviewed, while most studies describe a healthy environment for all people with ID (Vlot-Van Anrooij et al. 2020a), few focus specifically on adolescents with ID, and as such, this study addresses this population gap in the literature. Furthermore, this study describes this healthy environment from a South African perspective, complementing studies conducted in Europe.

### Problem statement

Despite acknowledging that a healthy environment promotes the well-being for adolescents with ID (Nielsen et al. 2021), and the mandates in statutes such as the *Constitution* of the Republic of South Africa, Rights of Patients Charter (NDoH 2002), there remain significant clinical practice and public health concerns regarding the provision of such healthy environments. The researcher noted the presence of unfavourable circumstances in healthcare facilities, including inadequate staffing, a lack of physical activities for ALWIDs, insufficient nutrition and deteriorating infrastructures. These challenges create unhealthy environments for adolescents. They require specialised care, and families face difficulties in providing care because of limitations in accessing the highest standard of quality care (Balcı et al. 2019; Mert & Köşgeroğlu 2022; Ntshingila et al. 2021). These limitations include limited services, insufficient equipment, inadequate funding and a lack of competent staff (Nielsen et al. 2021; Powers et al. 2021), all of which negatively impact healthcare delivery (Mimmo, Harrison & Hinchcliff 2018; Mimmo et al. 2019). This hinders the achievement of Goal 3’s targets to ensure universal health coverage and access to essential healthcare services (Carlsen & Bruggemann 2022; United Nations 2015). Furthermore, Vlot-Van Anrooij et al. (2020a) highlighted the fact that the characteristics of a healthy environment for people with ID are not well-understood.

The negative consequences of an unhealthy environment on adolescents with ID are evidenced through the Life Esidimeni tragedy where 1442 mental health patients were transferred from a long-term psychiatric hospital (Life Esidimeni) to other psychiatric hospitals (15%) or not-for-profit organisations (85%) resulting in the deaths of 131 patients between October 2015 and August 2017 (Makgoba 2016; Robertson & Makgoba 2018). Robertson and Makgoba (2018) in their findings on the Life Esidimeni tragedy, concluded that the high mortality rate was associated with the poor environment in the not-for-profit organisations to which some patients had been transferred. The deaths of 36 mental healthcare users provoked a public outcry. It is this evidence that influenced the researcher to explore nurse managers’ perspectives regarding the creation and promotion of a healthy environment for adolescents with ID to prevent another tragedy in South Africa. This emphasises the need for a healthy environment with a comprehensive range of quality services that cater to the needs of adolescents with IDs.

### Research aim

The purpose of this study is to describe nurse managers’ perspectives on the creation of a healthy environment for ALWIDs in health facilities.

### Research objectives

The study aims to explore nurse managers’ perspectives regarding the creation of a healthy environment for ALWIDs.

It also aims to recommend interventions that contribute to the creation of a healthy environment for ALWIDs based on the perspectives shared by nurse managers in this study.

## Research methods and design

### Study design

Explorative qualitative research seeks to gain insights into a situation, phenomenon, community or individual (Fouché, Strydom & Roestenburg 2021). Explorative qualitative research was used to enable the nurse managers to share their perspectives for a healthy environment that enables institutions that provide care for ALWIDs to achieve better health outcomes. Therefore, exploratory research was conducted to gain an understanding of the phenomena under study.

### Study setting

Three institutions directly involved in the care, treatment and rehabilitation of ALWIDs were included in the study. These institutions comprise a psychiatric institution, a public care and rehabilitation centre and a non-governmental organisation, all situated in the Tshwane district of Gauteng province. They were purposively chosen to acquire diverse and rich data.

### Population and sampling strategy

The study participants consisted of nurse managers from the three selected institutions in Tshwane district, South Africa, who are directly involved in the care, treatment and rehabilitation of adolescents with ID. Selecting nurse managers as participants ensured that the study captured the experiences, perspectives and expertise of individuals directly involved in managing nursing services. Purposive sampling, also known as convenience sampling, was used to intentionally select participants and sites that could provide valuable insights into the research problem being investigated. Nurse managers from the three institutions were purposively sampled to offer insights and rich data that contribute to the creation of a healthy environment for ALWIDs. Nurse managers who were willing to be interviewed participated in the study.

Thirteen nurse service managers from the three health institutions in Tshwane district participated in the study. The total pool included 15 nurse managers from these institutions: Institution 1 had one nurse manager, Institution 2 had two nurse managers and Institution 3 had 13 nurse managers. However, two nurse managers declined to be interviewed without providing reasons.

To recruit participants, the researcher emailed an information leaflet and consent form to the Chief Executive Officers and Clinical Education Training Units (CETU) for distribution to potential participants. Additionally, the researcher made a physical visit to the three institutions to recruit participants. Those willing to participate in the study signed the consent form. Recruitment occurred between May 2022 and June 2022, with follow-up appointments scheduled for data collection at convenient times for the participants.

### Inclusion criteria

Nurse managers who were willing to be interviewed participated in the study irrespective of gender and ethnic group. All nurse managers were proficient in English and preferred to be interviewed in English. This choice ensured uniformity in data collection and facilitated consistent data analysis throughout the study. The nurse managers selected were those working in institutions that provide care for adolescents with IDs and had at least 2 years of experience in this field.

### Exclusion criteria

Nurse managers who did not give voluntary consent to participate and those not working in institutions that cater to adolescents with IDs were excluded from this study. Similarly, nurse managers with less than 2 years of working experience were excluded. This decision was made because their limited experience might not have provided them with sufficient insights to contribute meaningfully to the objectives of this research.

### Data collection

Data were collected from June 2022 to August 2022 using individual semi-structured interviews, a method that, in qualitative studies, considers the phenomenon and the population of interest (Polit & Beck 2021). Face-to-face and telephonic interviews were conducted in English and lasted between 30 min and 60 min. An interview guide was used to facilitate the interview process. Interviews were recorded using a digital recorder to ensure no information was overlooked or omitted and to guarantee comprehensive documentation. All nurse managers could read and understand English. Many participants in the sample were unmarried women with bachelor’s degrees, aged between 42 and 64 years. Nine were females and four were males. Their work experience ranged from 10 to 39 years. The majority, eight, participated in telephonic interviews, while five interviews were conducted face-to-face in the nurse managers’ offices. To ensure privacy, a no-noise sign was placed on the door to avoid distractions. Coronavirus disease 2019 (COVID-19) protocols were followed during face-to-face interviews, including maintaining a 1.5-m social distance, wearing masks and providing hand sanitiser for hand hygiene. Instruments were also sanitised before use.

Open-ended questions were posed to nurse managers concerning their perspectives on creating a healthy environment for adolescents with intellectual disabilities. Two overarching exploratory questions were asked:

1. What is your perception regarding the creation of a healthy environment for ALWIDs?
2. What interventions would you recommend for the creation of a healthy environment for ALWIDs?

To ensure consistency and accuracy during data collection, a pilot testing phase was conducted; the results of the pilot testing were not included as part of the main findings. The data-collection instrument was carefully evaluated and deemed appropriate for the target population.

### Data analysis

The study employed Braun and Clarke’s thematic analysis, a six-step method for interpreting data and deriving thematic insights (Kiger & Varpio 2020). Initially, all recorded interviews were meticulously transcribed to capture participants’ perspectives accurately. Subsequently, the transcripts underwent iterative reviews to achieve familiarity with the data. The themes were identified through a process of organising and grouping related information, facilitated by an independent coder to enhance reliability. Consensus discussions were integral in refining themes and subthemes ensuring alignment with the data. Quotes from participants were integrated with themes to support their interpretation, facilitating the identification of meaningful patterns and insights within the dataset.

### Ethical considerations

Ethical clearance approval was granted by the University of South Africa College of Human Sciences (CHS) Research Ethics committee Rec: 240816-052 and Ref 90428595-CREC-CHS-2022 valid from 25 April 2022 to 25 April 2025. Participants gave verbal and written informed consent to participate in the study. Participation was voluntary, and opportunities were given to participants to withdraw at any given time without giving the reasons for their withdrawal. They were given the freedom to choose whether to use face-to-face or telephonic interviews. Numbers were used to protect the participants’ identities in the interest of promoting anonymity. Collected data were securely stored with encryption and password protection, limiting access to unauthorised individuals. Coronavirus disease 2019 protocols were observed during face-to-face interviews which included the wearing of masks, maintaining social distancing, sanitising hands and equipment and ensuring that the offices were cleaned before use.

## Results

Many participants were females, aged between 50 and 59 years, and had psychiatric training with over 10 years of professional experience. Table 1 shows a detailed demographic profile.

**TABLE 1:** Demographic characteristics (*N* = 13).

Participants’ demographics	Frequency	%
**Age group (years)**		
40–49	3	23.00
50–59	9	69.00
60–69	1	8.00
**Gender**		
Female	9	69.00
Male	4	31.00
**Highest qualifications**		
Diploma in General Nursing	1	8.00
Diploma in Nursing (General, Psychiatry and Community) and Midwifery	2	15.00
Post Graduate Diploma in Nursing Admin	1	8.00
BCur Degree nursing	5	38.00
Masters in Psychiatric nursing	4	31.00
**Area of employment**		
Public	12	92.00
Non-Governmental Organisation	1	8.00
**Years of nursing experience**		
10–19	6	46.00
20–29	5	38.00
30–39	2	16.00
**Rank**		
Operational manager	12	92.00
Deputy Director	1	8.00
**Number of institutions**		
Non-Governmental organisation: Institution 1	1	33.33
Psychiatric institution: Institution 2	1	33.33
Rehabilitation centre: Institution 3	1	33.33

On thematic analysis, four themes emerged: safe environment, collaborative approach, competent and adequate human resources, a conducive physical environment to provide the structural support and material and financial support. Table 2 provides the four themes that emerged.

**TABLE 2:** Emerged themes.

Themes	Subthemes
1. Safe environment	1.1 Free from harm and exploitation 1.2 Home-like environment 1.3 An environment promoting a trusting relationship of care 1.4 Access to quality patient care 1.5 Sense of enjoyment
2. Collaborative approach	2.1 User involvement 2.2 Family involvement and support
3. Competent and adequate staff	3.1 Competent staff 3.2 Adequate staff
4. Conducive physical environment to provide the structural support	4.1 Material and financial support

### Theme 1: Safe environment

Most participants emphasised the importance of providing a safe environment to prevent adolescents from exposure to harm, illnesses, obesity and premature death. A safe environment was seen as crucial for achieving positive patient outcomes, ensuring their safety, supporting their recovery and establishing a conducive working atmosphere for healthcare staff (Kneisley 2024; Maddineshat et al. 2024). Additionally, six subthemes emerged that underscored the need for a safe environment. The following statements reflect the participants’ narrative statements regarding a safe environment for individuals with intellectual disabilities:

‘We regard rehabilitation centres as a safe environment for them as they are still trying to find themselves like any other adolescent but because of their level of understanding and shortfalls, people in the society tend to take advantage of that.’ (P1F, Inst 2, 53-years-old)

‘Creation of a healthy environment should take into consideration various aspects such as ensuring the environment is safe, no hazards around and the users are always around people. The staff need to be vigilant.’ (P11F, Inst 2, 51-years-old)

‘The adolescents must be put in a safe place, not in cages but safe environment.’ (P13F, Inst 3, 53-years-old)

#### Subtheme 1.1: Free from harm and exploitation

The first subtheme to emerge was that adolescents with IDs must be free from harm and exploitation. The interview quotes listed in the subsequent text capture the narrative statements of the participants on this:

‘These individuals are vulnerable and subjected to abuse from community members and from social network platforms.’ (P1F, Inst 2, 53-years-old)

‘We need to provide an environment that is free from dangers to ensure normal development. They should not just be locked up. Mentally disabled adolescents must be treated the same as normal adolescents, but they must be protected from harmful things.’ (P4F, Inst 3, 64-years-old)

‘We need a hazard-free environment.’ (P5M, Inst 3, 50-years-old male)

‘A conducive environment free from harm is important for intellectually disabled adolescents so that they can talk and walk freely.’ (P6F, Inst 3, 57-years-old)

#### Subtheme 1.2: Home-like environment

Another perspective on a conducive environment for adolescents with ID is that long-term care institutions should feel like home. Quotes from two participants explain this theme:

‘The environment must be homely. Our institutions should be a home where they are happy.’ (P1F, Inst 2, 53-years-old)

‘The way the environment is created must be warm. Users will feel that it’s an environment fit for them, and they will be proud of the environment and be receptive.’ (P12M, Inst 3, 49-years-old)

#### Subtheme 1.3: An environment promoting a trusting relationship of care

Adolescents with ID must be in an atmosphere that they can trust to provide care. Two participants expressed this perspective. This is reflected by the narratives discussed further in the text:

‘We also need to build a rapport to promote a trusting relationship with our patients and all stakeholders.’ (P6F, Inst 3, 57-years-old female)

‘The environment must have a warm and relaxed atmosphere in which relationships are built on trust and mutual respect. The communication style must be sensitive and respectful even when things are tough.’ (P12M, Inst 3, 49-years-old)

#### Subtheme 1.4: Access to quality patient care

Most participants considered that access to quality patient care should be prioritised and given more attention. Nurse managers needed to play an advocacy role to ensure that adolescents with IDs access patient care. The thoughts of the participants about access to quality patient care follow:

‘We must be able to provide all the basic needs for them according to Maslow’s hierarchy of needs like self-actualisation, self-esteem and freedom of speech, where they are comfortable to be themselves which in turn will build their confidence levels. Physiological needs like food, water, security, and sanitation will ensure that the environment is safe.’ (P1F, Inst 2, 53-years-old)

‘A clean environment with access to fresh air, sunlight, healthy food and medication.’ (P2F, Inst 1, a 50-years-old)

‘I need to make sure that patients get food, clothes and the environment is clean.’ (P5M, Inst 3, 50-years-old)

‘It’s to give care to patients, their families are unable to take care of them because intellectually disabled patients are fully dependent on environmental care. They have bipolar and depression especially patients that don’t receive support from their families. When they’re depressed, they are throwing tantrums and harm themselves. We have a significant number of them who have psychotic disorders. You find its ID and psychotic disorders which they also develop. A full-time psychiatrist is also needed to treat the psychotic disorders. In my career, I have noted that mental or psychiatric hospitals are being ignored. The most attention is given to medical and surgical health issues. So, I would like the higher authority to take cognisance of mental health services because a human being thrives from being mentally stable.’ (P3M, Inst 3, 52-years-old)

‘As we work with mentally disabled patients, we are their eyes & ears. We must make sure that our patients are getting good care, the ward is conducive and free from smell or damaged items. When we go to patients the first thing is to bathe them and assess the patient, especially in the morning after shift change. The patient and the environment they live in must correlate. My role is to make sure that patients are getting proper health care. Because at the end of the day, they must bathe and eat. I must be a role model to my subordinates and lead by example.’ (P7F, Inst 3, 52-years-old)

‘Nurses need to fulfil the task of taking care of patients. Some difficult patients will need nurses that will provide tender care to them. I do the rounds every day to ensure the right care is being given to our patients. Intervene where there are challenges and give support and motivation to nurses.’ (P9F, Inst 3, 47-years-old)

‘First and foremost, I must advocate for a safe nursing area, an area that accommodates their needs. I must ensure that I protect their rights.’ (P10M, Inst 3, 54-years-old)

#### Subtheme 1.5: Sense of enjoyment

Six participants responded that a sense of pleasure is of key help for the adolescents to develop a positive self-concept. The following extracts are from participants’ narrative statements:

‘I and my staff should also foster a healthy environment so that these children can experience the joy they deserve. They must be provided with healthy food and have activities outside with music.’ (P2F, Inst 1, 50-years-old)

‘They need to be taken out regularly for entertainment. We should not trigger frustrations in our patients by treating them like adults because of how they look while forgetting their mental disability.’ (P3M, Inst 3, 52-years-old)

‘Creating a positive environment by having music classes and playing games to stimulate their abilities and be therapeutic. Having beautiful colours in the environment and making sure that it is clean. Soft colours to be considered would be grey, and purple. Not too much brightness. Having TV as well will form part of the therapeutic environment for them.’ (P8F, Inst 3, 42-years-old)

### Theme 2: Collaborative approach

The second theme to emerge was the collaboration with multi-disciplinary teams. This approach emphasises fostering cooperation among all stakeholders, including parents of adolescents with intellectual disabilities and healthcare professionals. The goal is to collectively address challenges and actively participate in reducing the burden of care (Ntshingila et al. 2021). This issue was described by participants as requiring support from social workers, occupational therapists and dieticians. Two subthemes emerged: the first was that the multi-disciplinary team must involve adolescents with ID and their family members. The quotes discussed in the text that follows illustrate this issue:

‘Support from other members of the multi-disciplinary team such as the social workers who will engage with families who leave their children at centres. Psychologists will come to assist with intellectually disabled adolescents’ emotions, and occupational therapists will assist with relevant activities.’ (P1F, Inst 2, 53-years-old)

‘The dietician is needed, they will need proper nutrition, if that is not well taken care of patients can develop irritations. Support from social workers, occupational therapists, and family members.’ (P9F, Inst 3, 47-years-old)

‘Nurses and occupational therapists are needed because users must be occupied all the time.’ (P11F, Inst 2, 51-years-old)

#### Subtheme 2.1: User involvement

Participants reported that users need to be involved in their care, treatment and rehabilitation. This was supported by the following statements:

‘Their social needs should be considered to help with building friendships with other people in their space.’ (P1F, Inst 2, 53-years-old)

‘Adolescents are never settled, and training on how to deal with their energy levels is crucial. Users need to be occupied and actively involved so that they positively use their energy.’ (P13F, Inst 3, 53-years-old)

‘My perception is that a healthy environment must help the users to develop a positive self-concept. Users must live in an environment that is safe and they must feel included to avoid developing mental difficulties. A healthy environment must be planned and set up in a way that supports positive relationships and good communication so that users are free to voice out their frustrations. The way the environment is created must be warm. Users will feel that it’s an environment fit for them, and they will be proud of the environment and be receptive.’ (P12M, Inst 3, 49-years-old)

#### Subtheme 2.2: Family involvement and support

Participants pronounced that parental support should be taken into consideration as evidenced by the following statements:

‘Input and support from family members. They need to engage with their families. They leave their children at the centres without any visits.’ (P1F, Inst 2, 53-years old)

‘Families not visiting the patients, the involvement of family is the most important thing ever.’ (P9F, Inst 3, 47-years-old)

‘The family as well if they can fully come on board and not take long to visit.’ (P8F, Inst 3, 42-years-old)

### Theme 3: Competent and adequate staff

Adolescents with IDs require sufficient competent staff to ensure comprehensive care. Nielsen et al. (2021) underscored the fact that competent personnel possess the requisite knowledge, skills, attitudes and values of self-determination and empowerment. This enables them to transform the environment, drive change and influence policies thereby playing a pivotal role in promoting health for individuals with mental and intellectual disabilities. Participants in this study highlighted the necessity of having adequate staff who are knowledgeable and passionate about caring for adolescents with IDs to promote a healthy environment. Some excerpts reflecting participants’ views on this matter are discussed in the text that follows.

#### Subtheme 3.1: Competent staff

‘My role is to ensure that I have competent staff who can manage the adolescents with understanding and compassion. As practitioners, we need to be vocal in terms of advocating for our users because they are the most vulnerable population in our society.’ (P1F, Inst 2, 53-years-old)

‘Skilled people are needed to come and assist.’ (P5M, Inst 3, 50-years-old)

‘My role is to motivate staff and others to perform their task to the best of their ability.’ (P6F, Inst 3, 57-years-old)

‘People that are working with these patients must be well-trained and understand their conditions. Hiring staff members with qualifications. A qualified dietitian is also important for our users who will understand the type of food our users need.’ (P8F, Inst 3, 42-years-old)

‘We need to have skilful nurses that will provide quality care and who are positive in mind.’ (P9F, Inst 3, 47-years-old)

#### Subtheme 3.2: Adequate staff

In addition to having competent staff, the participants also noted that adequate staff was necessary to provide a conducive environment for adolescents with ID. The text that follows lists some of the participants’ quotes in response to adequate staff:

‘We don’t get to be financed well in our institutions; our budgets are always cut. We are short of work staff.’ (P10M, Inst 3, 54-years-old)

‘Firstly, the shortage of staff that’s making it difficult to create a healthy environment for users. Nursing staff because they are the ones who are mostly in the company of the users. We need enrolled nurses and assistant nurses mostly as the professional nurses are busy with preparing medication and planning.’ (P12, Inst 3, 49-years-old)

### Theme 4: Conducive physical environment to provide the structural support

Participants also stressed that the physical environment plays a crucial role in supporting the care of adolescents with ID. Preconditions for an optimal physical environment include access to healthcare professionals, preventative measures, financial considerations and opportunities for engagement (Vlot-Van Anrooij et al. 2020b). Participants highlighted the importance of spacious and safe buildings capable of accommodating all necessary activities for adolescents with ID. The quotes illustrate their perspectives on the issue of structural support:

‘Infrastructure should be appropriate for the mental level of our patients. Constant renovation and change are needed because they are destructive.’ (P3M, Inst 3, 52-years-old)

‘The structure needs to be user-friendly. The structure must have all the necessary things such as a courtyard and a hazard-free environment.’ (P5M, Inst 3, 50-years-old)

‘The building is dilapidated with a lack of resources and toys. New buildings will do us good which are spacious because the ones we use are small and have an in-house occupational therapist department. New buildings should be designed specifically for this type of user. If they can maybe change the building to relate to their conditions because now, we are just improvising.’ (P6F, Inst 3, 57-years-old)

‘The building needs to be spacious and safe for them to be free. In most cases, we don’t get this thing because employers always say no funds.’ (P10M, Inst 3, 54-years-old)

#### Subtheme 4.1: Material and financial support

Participants reported that material and financial support should be taken into consideration when seeking to provide structural support for adolescents with ID. This was supported by the quotes discussed in the text that follows:

‘We do not have enough food as it is! I can be happy if businessmen around here can at least donate food for children. This place is partially subsidised by the DoH and other donors here and there. At times donors are scarce and COVID-19 disrupted a lot of donors. We’re not sure if others will return and donate for us. If there can be that good Samaritan who can donate some funds specifically for transport, petrol is the main challenge.’ (P2F, Inst 1, 50-years-old)

‘Lack of resources like budget constraints.’ (P1F, Inst 2, 53-years-old)

‘So, our budget is not enough to can cater for all specialised work, we are not yet categorised by the treasury as a level 3 specialised institution. So, in our budget, we devise some means to get specialists on sessions. Our directorate which is our authority, expects us to perform, and the budget is failing us. If maybe, we can be recognised as a tertiary institution, our budget might be increased. This will help to recruit specialists on a full term. With an increased budget our service care will improve.’ (P3M, Inst 3, 52-years-old)

## Discussion

The study aimed to explore nurse managers’ perspectives on a healthy environment for ALWIDs in health facilities. The study found that nurse managers perceived a safe environment, a collaborative approach, competent and adequate staff and the physical environment for structural support as healthy for adolescents with ID.

### Safe environment

The study’s findings underscored several elements essential for creating a safe environment, for adolescents with intellectual disabilities. These include freedom from harm and exploitation, fostering a home-like atmosphere, nurturing trusting caregiver–patient relationships, ensuring access to high-quality healthcare and promoting a sense of enjoyment. Participants emphasised the importance of placing adolescents in settings that are not restrictive or confining, but rather resemble a comforting home environment conducive to happiness, relaxation and normal adolescent development. They highlighted the importance of maintaining a warm and relaxed atmosphere, where relationships are built on trust and respect, even in challenging circumstances, with staff remaining vigilant to ensure safety.

Nurse managers were noted as advocates for prioritising and improving access to quality patient care, emphasising the necessity of clean environments offering essentials such as fresh air, sunlight, sanitation, security, nutritious food, bathing facilities, appropriate clothing and healthcare services. Additionally, participants recommended creating visually appealing and vibrant environments, using soft colours such as grey and purple, to enhance adolescents’ well-being. This includes providing access to toys, opportunities for games, music and television.

These perspectives align with the research by Vlot-Van Anrooij et al. (2020a), which emphasised creating aesthetically pleasing environments with ample natural light, secure spaces for physical activities and unobstructed access to healthcare services. Furthermore, these efforts correspond with the guidelines from the NDoH in 2003, emphasising the establishment of healthy environments with inclusive facilities that promote social interaction and meet the fundamental needs of children and adolescents. It is, therefore, important that the structural buildings should be accommodative so that their basic needs are met. This assertion by NDoH (2003) is also supported by findings from this study, as participants noted that the physical environment should have adequate space and should be safe to accommodate the needs of the adolescents with ID. A study conducted in South Africa reached a similar conclusion, stating that active engagement and advocacy from various stakeholders, such as families, professionals and neighbours, are vital for safeguarding the well-being of children with intellectual disabilities (Modula & Sumbane 2022). However, the findings of this study specifically underscored the active participation of professional staff and family members, without necessarily involving neighbours.

Furthermore, sound family and peer relationships should be strengthened to maximise their productivity and development. Although interactions with others are strongly pronounced, the findings of this study highlight the importance of protecting them from vulnerability and exploitation as they do not have the necessary capacity to judge bad intentions. According to the previous literature review (Ginis et al. 2021), to advance the provision of quality care for people living with intellectual disabilities, a multi-disciplinary team of professionals should work together to meet their health needs and share knowledge about the promotion of physical activities. This is essential for well-being and relatedness as per the SDT framework.

### Collaborative approach

The participants in this study highlighted the significance of collaborating with a multi-disciplinary team, including social workers, occupational therapists, dieticians, nurses and family members, to ensure comprehensive support for the care, treatment and rehabilitation of adolescents. They emphasised the importance of addressing social needs by establishing networks with families and individuals within their social circles. It was underscored that involving family members is crucial, as many are currently not actively involved, leaving the adolescents without regular visits or support. The active engagement of a multi-disciplinary team remains essential in creating a healthy environment for adolescents with intellectual disabilities. Roy et al. (2021) indicated that psychiatrists need to work together with nurses, clinical psychologists, advocates, therapists and families to improve service delivery to persons with ID/intellectual developmental disorders. Nurses should maintain continuous involvement in supporting families with intellectually disabled children at clinics, rehabilitation centres and within the community (Balcı et al. 2019). A study conducted in Uganda suggests that continued support and collaborative efforts can enhance the capacity of child and adolescent mental health services in similar developing countries such as South Africa (Rukundo et al. 2020). Furthermore, the World Psychiatric Association has set priorities to meet the needs of people with ID and to prevent the stigma related to mental illness. This can be achieved through partnerships with other professional organisations, non-governmental organisations and governmental agencies in the health, educational and social sectors. Supportive employment for youth with intellectual disabilities was found to enhance occupational opportunities, sustain work participation, address contextual barriers and uphold human rights (Engelbrecht, Van Niekerk & Shaw 2023). Significant changes in the social sector are required to support access to education, employment, housing and social benefits for people with mental health conditions and psychosocial disabilities (WHO 2021). Furthermore, scaling-up integrated, community-based mental health services is essential to achieve the changes advocated by the CRPD. This approach supports collaboration involving multi-disciplinary teams, and promotes the constructs of autonomy, competence and relatedness as outlined in SDT.

### Competent and adequate staff

The findings of this study highlighted the crucial role of well-trained and knowledgeable staff in meeting the needs of adolescents with intellectual disabilities. Competent staff members who demonstrate understanding, compassion, motivation, a positive mindset and advocacy skills are essential for providing high-quality care to this vulnerable population, who are among the most susceptible groups in society. To address the shortage of staff, which is of clinal concern, and hinders with the provision of a healthy environment, more nurses and assistant nurses need to be enrolled. This view was supported by Nielsen et al. (2021) and indicated that ALWIDs should have competent staff to provide for their complex healthcare needs. Therefore, they need competent staff to respond to their specific needs as they are dependent on others for services because of the complex nature of the disabilities. This fosters a sense of mastery and achievement, aligning with the competence and relatedness construct of SDT. The findings of this study also noted the issue of competent staff as described by Nielsen et al. (2021) in a study conducted in Australia. Conversely, a study conducted in Sydney by Ong et al. (2023) concluded that bringing about change among children and ALWIDs requires intrinsic motivation for change. Conversely, Smith et al. (2021) discussed that across the different modes of training, respondents identified accumulation of day-to-day professional experiences and their clinical experiences as most helpful; online modules were judged less helpful. This means that nurse managers should be motivated and willing to motivate others to come up with improvement strategies applicable to their settings and/or context. Day-to-day clinical and professional experience plays a major role to achieve positive outcomes.

### Conducive physical environment to provide the structural support

Study participants highlighted the critical need for material and financial support to overcome financial constraints, particularly exacerbated by factors such as the impact of COVID-19 on donor availability. There was a strong emphasis on increasing budget allocations and funding, especially to enhance specialist services and address transportation challenges participants recommended involving the business sector for financial support.

Research by Vlot-Van Anrooij et al. (2020a) emphasised the importance of adequate financial provisions to ensure access to nutritious food, engaging activities and resources for adaptation, all crucial elements for creating a supportive environment. Therefore, it is essential to reassess fund allocation and budgeting for healthcare institutions serving adolescents with intellectual disabilities, to improve their overall living conditions that promote good health.

### Strengths and limitations

The main strength of the study was to provide the perspectives of those nurse managers who are responsible for the care of adolescents with IDs. Perspectives from management enable policy influence, facilitating the provision of a conducive environment for adolescents with ID. The study was, however, limited to data from three institutions in the Tshwane district, and only nurse managers were included, with other healthcare providers being excluded from the study.

### Recommendations of the study

The study seeks to inform policymakers on what needs to be considered: address health disparities and enhance a healthy environment for ALWIDs. For nurse managers, the study implies that they should strive within their means for action to promote a conducive environment for ALWIDs such as ensuring adequate staff, promotion of physical activity of ALWIDs and competent staff through training. Nurse education should revise its curriculum to incorporate content that addresses the creation of supportive environments for ALWIDs in both theoretical learning and clinical practice. Additionally, there is a need for further research on the advocacy role of nurse managers within the healthcare system to enhance positive outcomes and maximise health for ALWIDs.

## Conclusion

This study highlights the components of a healthy environment necessary for ALWIDs in healthcare settings. A safe and supportive environment plays a foundational role in achieving quality care for these individuals. From the study findings, four overarching themes were drawn. The first theme concluded that a healthy environment for adolescents with ID should be safe. From their descriptions, this safe. environment should emulate a home environment, should be free from harm and exploitation and should also promote enjoyment and physical activity. The study also concluded that a conducive environment must involve a multi-disciplinary team that also includes family and the adolescents themselves with ID. Staff that provide care in long-term care facilities should also be competent and this can be assured by providing adequate training through in-service training to meet the needs of these adolescents. With regard to the physical environment, the study concluded that the buildings and surrounding environment should have adequate space to promote physical activity and provide specialised services such as occupational therapy. There is a need for financial and material support to provide this safe environment with adequate competent staff supported by a multi-disciplinary team and ample space. By doing this, progress can be made towards achieving the sustainable development goals (SDGs) and creating a more inclusive and sustainable society. Providing a safe environment with adequate resources and competent staff enables ALWIDs to exercise autonomy by participating in decision-making processes related to their care and well-being. This allows them to have a sense of agency and self-determination in their lives. This study addresses a significant gap and contributes to knowledge regarding the factors that enable the creation of a healthy environment, as perceived by nurse managers in a South African context. This knowledge is essential for improving service delivery and living conditions for ALWIDs.
